# Plasma for prevention and treatment of glycocalyx degradation in trauma and sepsis

**DOI:** 10.1186/s13054-024-05026-7

**Published:** 2024-07-20

**Authors:** M. S. Kravitz, N. Kattouf, I. J. Stewart, A. A. Ginde, E. P. Schmidt, N. I. Shapiro

**Affiliations:** 1 Department of Emergency Medicine, Beth Israel Deaconess Medical Center, Boston, MA USA; 2 Department of Emergency Medicine, Mount Sinai School of Medicine, New York, NY USA; 3 Department of Medicine, Uniformed Services University, Bethesda, MD USA; 4 Department of Emergency Medicine, University of Colorado School of Medicines, Aurora, CO USA; 5 Department of Medicine, Massachusetts General Hospital, Boston, MA USA

## Abstract

The endothelial glycocalyx, a gel-like layer that lines the luminal surface of blood vessels, is composed of proteoglycans, glycoproteins, and glycosaminoglycans. The endothelial glycocalyx plays an essential role in vascular homeostasis, and its degradation in trauma and sepsis can lead to microvascular dysfunction and organ injury. While there are no proven therapies for preventing or treating endothelial glycocalyx degradation, some initial literature suggests that plasma may have a therapeutic role in trauma and sepsis patients. Overall, the literature suggesting the use of plasma as a therapy for endothelial glycocalyx degradation is non-clinical basic science or exploratory. Plasma is an established therapy in the resuscitation of patients with hemorrhage for restoration of coagulation factors. However, plasma also contains other bioactive components, including sphingosine-1 phosphate, antithrombin, and adiponectin, which may protect and restore the endothelial glycocalyx, thereby helping to maintain or restore vascular homeostasis. This narrative review begins by describing the endothelial glycocalyx in health and disease: we discuss the overlapping disease mechanisms in trauma and sepsis that lead to its damage and introduce plasma transfusion as a potential therapy for prevention and treatment of endothelial glycocalyx degradation. Second, we review the literature on plasma as an exploratory therapy for endothelial glycocalyx degradation in trauma and sepsis. Third, we discuss the safety of plasma transfusion by reviewing the adverse events associated with plasma and other blood product transfusions, and we examine modern transfusion precautions that have enhanced the safety of plasma transfusion. We conclude that the literature proposes that plasma may have the potential to prevent and treat endothelial glycocalyx degradation in trauma and sepsis, indicating the need for further research.

**Supplementary Information:**

The online version contains supplementary material available at 10.1186/s13054-024-05026-7.

## Introduction

Trauma and sepsis are leading causes of morbidity and mortality [1–3]. Traumatic hemorrhage and sepsis share the common pathophysiology of endothelial glycocalyx (hereafter referred to as glycocalyx) degradation and endotheliopathy, for which plasma transfusion is emerging as a possible therapy, and additionally as preventative intervention that spares the glycocalyx from degradation [4–6]. Plasma transfusion is commonly used for the replenishment of coagulation factors and as a component of a balanced massive transfusion protocol in the setting of hemorrhage, alongside red blood cells and platelets [7–12]. Yet, plasma, the non-cellular component of blood, also contains numerous other biologically-active components that influence homeostatic and pathogenic pathways other than coagulation. These bioactive components, which include sphingosine-1 phosphate, antithrombin, and adiponectin, may prevent and restore damage to the glycocalyx, reduce endothelial cell permeability and leukocyte adhesion, and decrease inflammation in critical illnesses [13–22]. This narrative review aims to demonstrate the evidence for the hypothesis that plasma is a potential therapeutic, and glycocalyx sparing, agent in trauma and sepsis (beyond treatment of coagulopathy), through its potential protective and restorative effects on the glycocalyx.

## The role of the glycocalyx in health

**Fig. 1:**
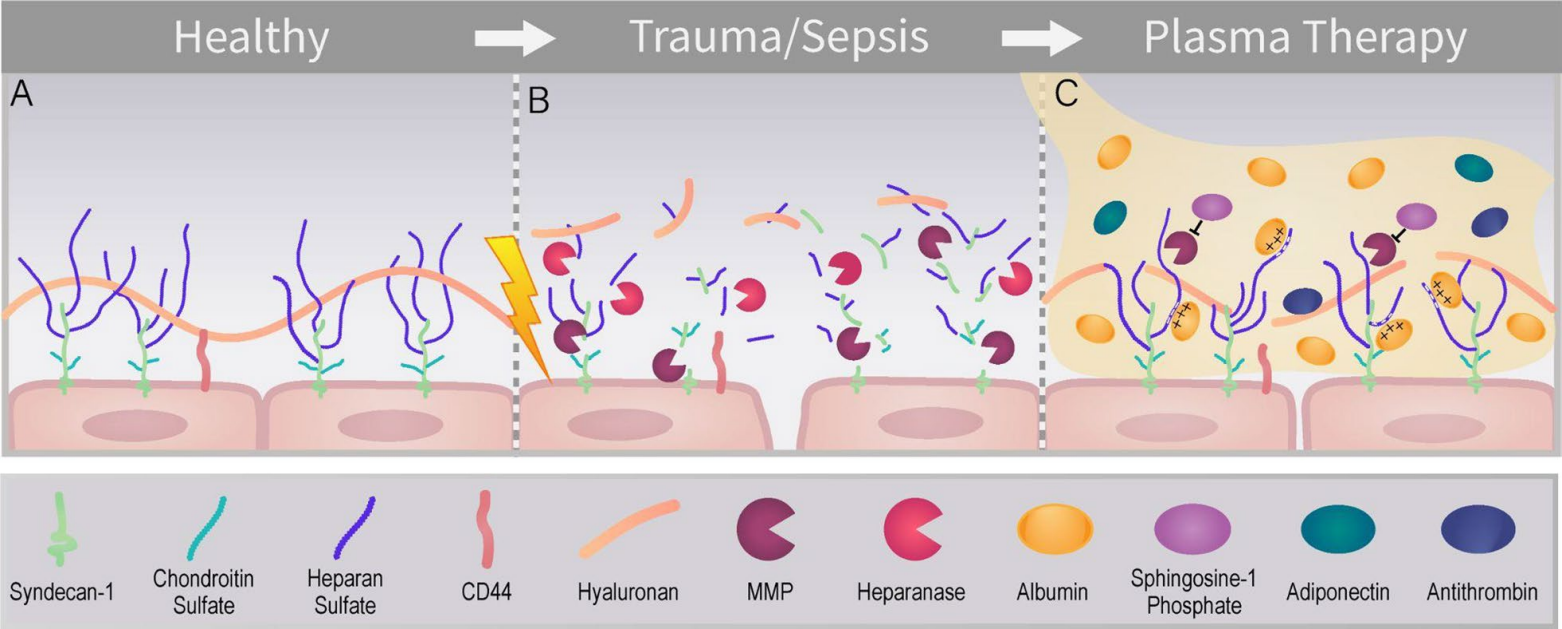
**A** The healthy endothelial glycocalyx. **B** Degradation of the endothelial glycocalyx caused by sympatho-adrenal hyperactivation pathophysiology seen in trauma and sepsis. This leads to endothelial cell activation, glycocalyx degradation, and vascular leakage. MMPs (matrix metalloproteinase) and heparanase are shown degrading the endothelial glycocalyx. **C** Potential mechanisms by which plasma transfusion can restore the damaged glycocalyx, including inhibition of MMPs by sphingosine-1 phosphate, the glycocalyx-stabilizing effect of albumin, and the endothelial-protective effects of adiponectin and antithrombin

The vascular endothelium forms the intimal surface of blood vessels [23]. The luminal surface of the endothelium is coated with the glycocalyx, an apical matrix composed of membrane bound proteoglycans and their associated glycosaminoglycans (GAGs), glycoproteins, and plasma proteins (Fig. 1) [5, 24]. Proteoglycans are glycosylphosphatidylinositol (GPI) anchored (e.g. glypicans) or transmembrane (e.g. syndecans) proteins that are covalently bound to sulfated glycosaminoglycans (such as heparan sulfate and chondroitin sulfate) which extend into the vascular lumen [25, 26]. By virtue of their negative charge, heparan sulfate and chondroitin sulfate electrostatically interact with plasma proteins [27]. Hyaluronan is not sulfated and sequesters water, which stabilizes the gel like structure, and binds to the glycoprotein CD44 [28]. Playing a crucial role in maintaining vascular health, the glycocalyx helps to regulate various homeostatic functions such as blood vessel tone, leukocyte adhesion to endothelial cells, blood clot formation, and maintenance of the endothelial barrier [5, 24, 29–35].

### Glycocalyx degradation in sepsis

Sepsis, the injurious systemic response to infection, often leads to significant endothelial cell activation and shedding of the glycocalyx (Fig. 1). During sepsis, the innate immune response triggers endothelial cells to release adhesion molecules, chemoattractants, vasoactive compounds, and inflammatory cytokines, occurring in parallel with sympathoadrenal hyperactivation [4, 36]. This leads to degradation of the endothelial glycocalyx through endothelial cell activation and the action of proteases, such as matrix metalloproteinases (MMPs), and glycosaminoglycan-degrading enzymes, such as heparanase [37–41]. This shedding contributes to the local microcirculatory dysfunction characteristic of sepsis, marked by vascular leak, tissue swelling, hypotension, and reduced oxygen supply to tissues, which leads to organ injury [36, 42].

### The overlapping pathophysiology of sepsis and trauma

Similar to sepsis, traumatic hemorrhage also involves a systemic disruptive inflammatory response that damages the glycocalyx, leading to organ dysfunction and worsening disease, which has been termed the Endotheliopathy of Trauma (EoT) [4, 6, 43, 44]. Both sepsis and trauma are syndromes characterized by sympatho-adrenal hyperactivation, leading to endothelial cell activation and glycocalyx degradation (endotheliopathy). This unifying pathophysiology of critical illness, termed Shock Induced Endotheliopathy (SHINE) by Johansson et al., is proposed to be conserved across acute critical illnesses and evolutionarily adapted [4]. SHINE is associated within glycocalyx shedding, capillary leakage, microvascular thrombosis, and organ failure [4]. Interestingly, trauma patients with endothelial glycocalyx degradation, as quantified by elevated circulating syndecan-1 levels after injury, have a higher likelihood of developing sepsis compared to those with normal syndecan-1 levels, suggesting convergent pathogenic mechanisms in these two critical illnesses characterized by endotheliopathy [45].

### Plasma transfusion as a potential therapy for endothelial glycocalyx degradation

The majority of the evidence in support of the hypothesis that plasma transfusion is a potential therapeutic agent for the treatment of glycocalyx degradation is derived primarily from studies of trauma, particularly in the context of hemorrhagic shock, where plasma is now a standard therapeutic intervention during massive transfusion [44]. In contrast, there is a relative scarcity of studies exploring plasma’s impact in sepsis. Given the shared pathophysiology of shock-induced sympatho-adrenal hyperactivation and subsequent endothelial cell activation and glycocalyx degradation across these critical illnesses, there are likely overlapping therapeutic mechanisms between the use of plasma in hemorrhagic shock and its application in other critical conditions [4]. This common pathway suggests that the benefits of plasma on the glycocalyx observed in trauma care could inform broader applications of plasma as a therapy in other critical illnesses characterized by endotheliopathy. Indeed, it is suggested that plasma is potentially beneficial in the treatment of non-hemorrhagic disease states with endotheliopathy [6]. This review will focus on studies of plasma’s impact on the glycocalyx in trauma and sepsis. Since the endotheliopathy of sepsis and trauma share similar pathophysiology, an intriguing hypothesis is that the potential benefits of plasma on the glycocalyx in trauma will translate to sepsis.

### The biological mechanism of plasma transfusion’s impact on the glycocalyx

Plasma contains numerous bioactive components, several of which have proposed interaction with the glycocalyx (Fig. 1). Albumin, while overall an anionic constituent of plasma, has positively charged arginine residues that are hypothesized to interact with the negatively charged glycosaminoglycans of the glycocalyx, leading to a pleiotropic stabilizing effect on the glycocalyx [46]. Glycocalyx stabilization by albumin and other plasma proteins such as the HDL-associated apolipoprotein M is additionally mediated by endothelial delivery of sphingosine-1-phosphate (S1P), a vascular-protective lipid shown to attenuate endothelial glycocalyx shedding by inhibiting MMPs [15, 41, 47–49]. The actual impact of albumin in plasma on glycocalyx degradation remains questionable due to the varying concentrations of albumin in fresh frozen plasma (FFP), which are slightly below physiologic levels. However, resuscitation with plasma would be much less dilutional to albumin levels than crystalloid resuscitation. Plasma serine proteases such as antithrombin and plasma adiponectin additionally may impart protective effects on glycocalyx integrity, but further research is needed to elucidate their mechanism of action [17, 50, 51].

### Plasma preparations

Plasma is available in various preparations, including FFP, thawed plasma, and dried plasma. FFP is frozen within eight hours of collection and thawed immediately prior to usage, and contains all clotting factors and proteins found in blood. Thawed plasma is previously frozen plasma that is stored at 1 to 6 °C for up to 5 days, but with a measurable decrease in FV and FVIII activity level [52–54]. Dried plasma includes lyophilized plasma and spray dried plasma, which have logistical advantages of not requiring freezing or refrigeration for storage [55, 56]. Unless specifically stated, throughout this review the term ‘plasma’ broadly refers to any of these preparations. Further studies are needed to determine whether different plasma preparations have similar levels of sphingosine-1-phosphate and other components that affect the glycocalyx, and if these variations impact clinical outcomes.

### Plasma transfusion as a potential therapy for glycocalyx degradation in trauma: pre-clinical literature

The potential utility of plasma as a glycocalyx-protective and restorative agent for treatment of endotheliopathy is supported by trauma models of hemorrhagic shock (Supplemental Table 1). Peng et al. used a mouse model of hemorrhagic shock which was resuscitated with either FFP or Lactated Ringers (LR). Endothelial permeability was significantly lower in mice resuscitated with FFP compared to those treated with LR. Furthermore, FFP resuscitation led to a reduction in inflammation as evidenced by markedly lower levels of myeloperoxidase immunofluorescence (an indicator of neutrophil infiltration) in the FFP-treated group compared to the LR group [20]. Pati et al. used an in vitro model of human pulmonary endothelial cells (PEC) treated in a hypoxic incubator to represent hypoxia from hemorrhagic shock and then treated with FFP or LR. FFP was found to be more effective at inhibiting PEC permeability compared to LR [57]. Kozar et al. conducted a study using a rat model of hemorrhagic shock, where the rats were resuscitated with either FFP or LR. The study found that resuscitation with FFP led to early signs of glycocalyx restoration, observable under electron microscopy, starting from around two hours post-resuscitation. This was in contrast to the LR group, where no glycocalyx restoration was noted. Additionally, the thickness of the glycocalyx post-treatment was significantly greater in the FFP group compared to the LR group. Furthermore, the study assessed the expression of syndecan-1 (a proteoglycan) mRNA in the lungs. Syndecan-1 mRNA expression in the lungs was significantly lower in rats exposed to hemorrhagic shock than control rats. Resuscitation with LR further decreased this mRNA expression. However, resuscitation with FFP restored syndecan-1 mRNA expression to levels comparable to the control group, suggesting activation of mechanisms driving glycocalyx reconstitution [19]. Additional preclinical studies demonstrate that resuscitation with FFP in hemorrhagic shock models reduces glycocalyx shedding, endothelial permeability, white blood cell adhesion, and endothelial cell apoptosis, and also improves vital signs and laboratory parameters such as blood pH, lactic acid, base excess, and respiratory rate [13, 18, 22, 58–60]. Overall these preclinical studies suggest benefits of FFP in trauma, independent of replenishment of coagulation factors.

### Plasma transfusion as a potential therapy for glycocalyx degradation in trauma: clinical literature

Expanding upon these basic science investigations, clinical studies have examined the administration of plasma to trauma patients (Supplemental Table 2). The literature supports plasma’s use for improving clinical outcomes in trauma [61–65]. However, there is a paucity of research examining the link between plasma’s impact on the glycocalyx to observed clinical outcomes. Therefore, there is insufficient data at this time to determine whether the link between the administration of plasma with clinical benefits is mediated through the glycocalyx.

One study that highlights the potential benefit of plasma on the endothelial glycocalyx in a clinical setting is a secondary analysis of the Prehospital Air Medical Plasma (PAMPer) trial [12, 66]. PAMPer showed a significant decrease in 30-day mortality for trauma patients at risk of hemorrhagic shock who were randomized to receive plasma compared to those who did not (23.2% vs. 33.0%, *p* < 0.03). In the secondary analysis of PAMPer, Gruen et al. assayed endothelial, glycocalyx and inflammatory markers from 405 trauma patients enrolled in this trial. Plasma administration was associated with a decrease in endothelial and glycocalyx biomarkers (syndecan-1, thrombomodulin, and vascular endothelial growth factor) and decreased pro-inflammatory mediators (IL-6, TNF-α, and MCP-1) in a subgroup of patients with greater injury severity scores and a higher incidence of blunt trauma. Further research is needed to examine if any part of the clinical benefits of plasma in trauma are mediated through the glycocalyx.

### Glycocalyx degradation in sepsis

Pre-clinical and clinical studies have established a key role for endothelial glycocalyx dysfunction in the pathophysiology of sepsis (Supplemental Tables 3 and 4). Additionally, increased levels of endothelial glycocalyx degradation products in blood samples correlate with the severity and mortality of sepsis [67–70]. There is also a link between sepsis-induced endothelial glycocalyx dysfunction and organ dysfunction, such as acute lung injury, acute respiratory distress syndrome, intestinal dysfunction, and acute kidney injury [37, 38, 71–73]. Considering the connections between endothelial glycocalyx dysfunction and increased morbidity, mortality, and organ dysfunction in sepsis, strategically targeting the endothelial glycocalyx is emerging as a potential therapeutic strategy in sepsis management.

### Plasma transfusion as a potential therapy for glycocalyx degradation in sepsis: pre-clinical literature

Based on the initial data in support of the hypothesis that plasma resuscitation improves outcomes in traumatic hemorrhagic shock in part through the attenuation of endothelial injury, which is pathophysiologically similar to the endothelial injury seen in sepsis, several basic science studies have examined the impact of plasma on endothelial glycocalyx degradation in sepsis (Supplemental Table 3). Chang et al. conducted a study comparing rat FFP versus normal saline resuscitation of a rat cecal ligation and puncture model of polymicrobial sepsis. Resuscitation with FFP in this study was associated with increased survival at 48 h [74]. FFP resuscitation, as opposed to normal saline resuscitation, was linked to a reduced increase in post-resuscitation blood levels of epinephrine, norepinephrine, IL-6, and syndecan-1. Furthermore, FFP resuscitation compared to normal saline resuscitation had significantly improved PO2/FiO2 ratio, and decreased lung wet-to-dry weight ratio, which suggests less pulmonary edema in the plasma resuscitation group [74]. Conversely, Barry et al. used a mouse model of sepsis and randomized mice to receive either lyophilized plasma or LR, and found no significant difference in mortality or post resuscitation plasma biomarkers of inflammation between treatment groups. However, lyophilized plasma resuscitation did induce a decrease in pulmonary inflammatory gene expression. While the work of Barry et al. seemingly contradicts that of Chang et al., several limitations in Barry et al.’s study need consideration, including the use of human lyophilized plasma in a mouse model, the use of lyophilized plasma instead FFP, and an additional freeze–thaw cycle of the lyophilized plasma; further research is therefore needed.

Sheck et al. studied the impact of FFP on the endothelial glycocalyx by measuring syndecan-1 levels of patients post-operative from major abdominal surgery who received plasma compared to those who did not receive FFP. Circulating syndecan-1 levels were lower in patients who underwent major abdominal surgery and were transfused with FFP compared to those who were not transfused. The authors complemented this observational human study by performing mechanistic studies using human pulmonary microvascular endothelial cells. In this model, FFP treatment attenuated LPS-induced pulmonary endothelial permeability in vitro, as assessed using a FITC-dextran transwell assay [75]. In addition, adding FFP to cells exposed to LPS was associated with a significant attenuation in endothelial activation, as demonstrated by a decrease in endothelial gene expression of ICAM-1, VCAM-1, and ANG-2 [75]. Unexpectedly, FFP treatment increased LPS-induced leukocyte adhesion to endothelial cells in vitro. Taken together, this study showed that FFP decreased biomarkers of endothelial glycocalyx degradation in post-operative patients, and showed that in an in vitro model of endothelial cells FFP exposure after LPS exposure improved endothelial cell barrier function [76].

### Plasma transfusion as a potential therapy for glycocalyx degradation in sepsis: clinical literature (or lack thereof)

The literature evaluating the clinical use of plasma as a therapy and restorative agent for glycocalyx degradation in human sepsis patients is quite limited (Supplemental Table 4), precluding any conclusion to be drawn. In a prospective substudy of a randomized clinical trial of 33 critically ill, non-bleeding coagulopathic patients, Straat et al. measured biomarkers of endothelial function and inflammatory cytokines before and after elective FFP transfusion prior to an invasive procedure [77]. About half of the patients in this cohort had sepsis (n = 15, 45%), and the median SOFA score was 11 (IQR 10–14). FFP administration led to a significant decrease in circulating TNF-ɑ (11.3 pg/ml vs 2.3 pg/ml, *p* < 0.01) and syndecan-1 levels (675 pg/ml vs 565 pg/ml, *p* < 0.01) [77]. These findings are hypothesis-generating that FFP administration decreases inflammation and glycocalyx shedding in non-bleeding, coagulopathic, critically ill patients. Studies examining the impact of plasma on clinical outcomes in sepsis show mixed results, are limited, and are inconclusive [78, 79].

### Safety of plasma transfusion

Adverse events reported with plasma transfusions include allergic reactions, transfusion associated lung injury (TRALI), transfusion associated circulatory overload (TACO), and infection [80, 81]. However, hemovigilance methods have made transfusions safer. The incidence of TRALI has decreased since the implementation of modern plasma collection techniques such as preferential use of male donor plasma and leukocyte antibody screening [82–84]. Pathogen inactivation methods such as solvent and detergent treatment and amotosalen plus UVA light have proven to be safe for inactivating numerous infectious pathogens from blood products [85, 86].

Plasma contains coagulation factors, and there is a risk that plasma transfusion could lead to thromboembolism. A retrospective study of 381 trauma patients receiving FFP and packed red blood cells (PRBCs) showed that each unit of FFP transfused increased the risk of venous thromboembolism in patients who received fewer than 4 units of PRBCs [87]. The Pragmatic Randomized Optimal Platelet and Plasma Ratios (PROPPR) trial transfused trauma patients with either a 1:1:1 or 1:1:2 ratio of plasma, platelets, and red blood cells. In this trial of 680 patients, those in the 1:1:1 group received more plasma than those in the 1:1:2 group (5 units vs. 7 units, *p* < 0.01). However, there was no increase in venous thromboembolism or acute respiratory distress syndrome between the groups [64]. Further research is needed to better understand the exact risk that plasma transfusion has on thromboembolism, and any patients enrolled in studies of plasma should be monitored for thromboembolism.

During the COVID-19 pandemic, convalescent plasma, which is FFP with specific antibodies from those convalescing from disease, was extensively studied as a potential therapy. A Cochrane systematic review and meta-analysis, which included 33 randomized controlled trials, indicates that plasma has little to no impact on the risk of serious adverse events with moderate certainty [88]. This Cochrane review also showed no mortality benefit from plasma. However, due to differences in the pathophysiologies between COVID-19 and bacterial sepsis, further research of plasma in the context of bacterial sepsis is warranted.

## Conclusion

In conclusion, this narrative review demonstrates that there is initial evidence supporting the hypothesis that plasma reduces glycocalyx degradation in pre-clinical studies and biomarkers in clinical studies of trauma and sepsis. Furthermore, this review concludes that, at this time, there is a lack of evidence to prove whether plasma’s impact on clinical outcomes is mediated through the glycocalyx. However, this remains an intriguing direction for future research. Although the majority of data in support of this hypothesis originates from trauma-related studies, the shared pathophysiology of sepsis and trauma suggests that plasma’s beneficial effects on the glycocalyx in trauma might extend to sepsis. Further research, especially in the context of sepsis, is needed to fully understand the therapeutic potential and risks of plasma before it is used clinically for the treatment of glycocalyx degradation. It is also crucial to further study S1P, albumin, antithrombin, and adiponectin, to further elucidate the biologic mechanisms through which these components impact the glycocalyx, and to explore methods for enriching these components.

### Supplementary Information

Supplementary Material 1.
