# Supplementary material for: Plasma for prevention and treatment of glycocalyx degradation in trauma and sepsis
Source: Crit Care. 2024 Jul 20;28:254. doi: 10.1186/s13054-024-05026-7 (PMC11265047; doi:10.1186/s13054-024-05026-7)
Supplement: Supplementary file 1 — Supplementary Material 1. [file 13054_2024_5026_MOESM1_ESM.pdf]

Supplemental Table 1: Selected Preclinical Studies of Plasma in Trauma

Authors	Year	Experiment	Outcome(s)	Findings
Lopez et al. [16]	2020	ATIII and syndecan-1 levels measured in severely injured trauma patients (N=125)<br><br>In vitro HLMVEC were pretreated with FFP, ATIII deficient FFP, or purified ATIII followed by TNF-α stimulation<br><br>Male mice were subjected to fixed pressure exsanguination model of hemorrhagic shock followed by resuscitation with FFP, ATIII deficient FFP, or ATIII deficient FFP with ATIII repletion	Syndecan-1<br><br>Endothelial barrier function	Pearson correlation analysis of syndecan-1 and antithrombin III (ATIII) levels, measured on the third day of hospital stay, revealed a significant negative correlation. This indicates that higher levels of syndecan-1 are associated with lower levels of ATIII .(R = -0.62; P < 0.0001).<br><br>In an in vitro model of HVLMEC FFP and ATIII prevented TNF-α induced permeability (P < 0.05), and ATIII deficient FFP had no effect. ATIII restoration reestablished its protective effects in a dose-dependent manner.<br><br>In mice syndecan-1 expression was increased following FFP resuscitation and no improvement in syndecan-1 expression was seen with ATIII deficient FFP.
Pati et al.[89]	2018	In vitro study<br><br>Cultured endothelial cells treated with either fresh frozen plasma or lyophilized plasma<br><br>In vivo hemorrhagic shock mouse model treated with either FFP or lyophilized plasma.	Endothelial permeability<br><br>Trans endothelial resistance<br><br>White blood cell binding	In the in vitro studies, FFP and lyophilized plasma had similar protective effects on the endothelial cells. They both similarly decreased endothelial cell permeability, increased transendothelial resistance, decreased white blood cell binding and maintained the adherens junctions.<br><br>In the in vivo studies, FFP and lyophilized plasma similarly reduced injury, vascular leak and inflammation in the lungs.

Authors	Year	Experiment	Outcome(s)	Findings
			Adherens junctions preservation	
Diebel et al.[90]	2018	Human umbilical vein endothelial cells were treated with epinephrine and hypoxia.<br>They were then treated with either media alone or human plasma.	Glycocalyx injury<br><br>Syndecan-1 level<br><br>Hyaluronic acid level	Treatment of human umbilical cells with FFP after exposure to hypoxia mitigated glycocalyx degradation (this was proven by maintaining glycocalyx thickness under fluorescent microscopy after treatment with FFP post hypoxia).<br><br>Treatment of human umbilical cells with FFP post hypoxia returned syndecan-1 and hyaluronic acid to control levels (preinjury levels).
Nelson et al.[91]	2016	Rats exposed to hemorrhagic shock then resuscitated with FFP or ringer's acetate	Heparan sulfate level	Resuscitation with ringer's acetate led to a higher heparan sulfate level (92 ug/ml) compared to resuscitation with FFP (53 ug/ml).

Authors	Year	Experiment	Outcome(s)	Findings
Potter et al. [92]	2015	Hemorrhagic shock rat model followed by resuscitation with FFP or lactated ringer.	Endothelial monolayer resistance<br><br>VE-cadherin and B-catenin<br><br>White blood cell infiltration<br><br>Endothelial adherens and tight junctions	Resuscitation with FFP after shock decreased endothelial permeability by enhancing endothelial monolayer resistance. This improvement was due to the restoration of VE-cadherin and β-catenin in the endothelial cells post-FFP treatment, an effect not observed with lactated Ringer's resuscitation.<br><br>Additionally, FFP inhibited WBC infiltration, reduced degradation of endothelial adherens and tight junctions, and corrected MAP and base excess induced by hemorrhagic shock.
Peng et al.[20]	2013	In vivo hemorrhagic shock rat model<br><br>Rats were resuscitated with either lactated ringer or FFP	Pulmonary endothelial permeability and neutrophil infiltration	Pulmonary endothelial permeability, measured by extravasation of intravenously delivered fluorescently conjugated dextran using an In Vivo Imaging System, was reduced with FFP compared to lactated ringers ($4.1 \times 10^{10} \pm 3.2 \times 10^{9}$ vs $6.7 \times 10^{10} \pm 5.0 \times 10^{9}$ respectively).<br><br>Myeloperoxidase immunofluorescence (a measure of neutrophil infiltration) was significantly lower in the FFP group compared to the lactated ringer group (485±65 RFUs vs 815±126 RFUs respectively)<br><br>Treatment with FFP decreased syndecan-1 shedding compared to lactated ringer (103 ng/ml vs 55 ng/ml)

Authors	Year	Experiment	Outcome(s)	Findings
Torres et al.[13]	2013	Rat model evaluating cremaster muscle microvessel glycocalyx thickness after 40% hemorrhage and resuscitation with either lactated ringer, FFP or Hextend.	Syndecan-1	Syndecan-1 levels were significantly higher after resuscitation with lactated Ringer or Hextend compared to FFP, showing about a 160% increase from baseline, versus an 80% increase with FFP. Despite this, all resuscitation methods—lactated Ringer, Hextend, and FFP—improved blood pH, lactic acid, base excess, and respiratory rate, with FFP yielding the most significant recovery.
Haywood et al.[18]	2011	In vitro model of endothelial injury.<br><br>Resuscitation with FFP	Syndecan-1 level and endothelial permeability	In vitro, endothelial permeability significantly increased following injury when compared to normal controls (15,916 RFU[††] vs. 4,898 RFU).<br><br>Resuscitation with Fresh Frozen Plasma (FFP) after endothelial injury returned permeability to normal levels (3,268 RFU).<br><br>Syndecan-1 immunostaining decreased after endothelial injury but was restored to higher levels upon resuscitation with FFP (114 RFU vs. 228 RFU, respectively).
Kozar et al.[19]	2011	Rats subjected to hemorrhagic shock then resuscitated with either lactated ringer or FFP	Glycocalyx thickness<br><br>Syndecan-1 mRNA expression	Resuscitation with FFP showed early signs of glycocalyx restoration (starting 2 hours post-resuscitation) under electron microscopy as compared to resuscitation with lactated ringer which did not show glycocalyx restoration.<br><br>Syndecan-1 mRNA expression in the lungs was significantly lower in the hemorrhagic shock group compared to the control group (1.39 ± 0.22 vs 3.03 ± 0.22, $p<0.02$).<br><br>Lactated ringer resuscitation after hemorrhagic shock further decreased syndecan-1 mRNA (0.82 ± 0.03, $p<0.001$). Resuscitation with plasma restored mRNA expression to 2.76 ± 0.03.

Fresh Frozen Plasma (FFP), Tumor necrosis factor alpha (TNF-α), Antithrombin-III (ATIII), Human Lung Microvascular Endothelial Cells (HLMVEC), Relative Fluorescence Units

Supplemental Table 2: Selected Clinical Studies of Plasma in Trauma

Authors	Year	Population	n	Design	Outcome	Result
Gruen et al. [66]	2020	Prehospital trauma patients who were enrolled in the Prehospital Air Medical Plasma trial RCT (FFP* versus control).	405	Secondary biomarker analysis of PAMPer	Endothelial and inflammatory markers	Plasma administration was associated with a decrease in endothelial damage markers (syndecan-1, thrombomodulin and vascular endothelial growth factor) and decreased pro-inflammatory mediators (IL-6, TNF-α and MCP-1) in a subgroup of patients with greater injury severity and a higher incidence of blunt trauma.
Sperry et al. (PAMPer) [12]	2018	Pre-hospital trauma patients.	501	Multicenter randomized clinical trial<br><br>Intervention: prehospital plasma therapy versus standard of care	Survival	Prehospital administration of plasma group had decreased 30-day mortality (23.2% vs 33.0%, difference= -9.8% 95% CI= -18.6 to -1.0%, p=0.03)
Moore et al.[93]	2018	Pre-hospital trauma patients	144	Single Center Randomized Clinical Trial	Survival	Prehospital administration of plasma was not associated with a difference in mortality at 28 days (15% in the plasma group and 10% in control group, p =0.37).
Spinella et al.[61]	2008	Trauma patients admitted to a combat support hospital of patients who received at least a unit of blood product.	708	Retrospective study	Survival	Each unit of FFP transfused was associated with increased survival (OR=1.17 95% CI= 1.06-1.9, p=0.002).
Holcomb et al.[9]	2008	Civilian trauma patients who received ≥ 10 units of RBCs within the first 24 hours.	466	Retrospective chart review	Survival	30 day survival was significantly higher in the high plasma to RBC ratio group (>1:2) compared to the low plasma to RBC ratio group (<1:2) (59.6 % vs 40.4%, p<0.01 respectively).

Borgman et al.[8]	2007		246	Retrospective chart review of 246 trauma patients admitted to a US army combat support hospital and received ≥ 10 units of RBCs.	Survival	The plasma to RBC[‡] ratio was independently associated with survival (OR=8.6 95 % CI= 2.1-35.2).

Fresh frozen plasma (FFP), Tumor necrosis factor alpha (TNF-ɑ), Monocyte Chemoattractant Protein-1 (MCP-1), Red blood cells (RBC)

Supplemental Table 3: Preclinical Studies of Plasma in Sepsis

Authors	Year	Design	Outcome	Result
Barry et al[76]	2022	Male mice received a peritoneal cecal slurry injection<br><br>Lyophilized plasma versus lactated ringer resuscitation	Mortality<br><br>Bronchoalveolar lavage total protein concentration (indicator of permeability)<br><br>Biomarkers of inflammation and endothelial function	There was no difference in short term mortality between groups (38% LR group vs 47% plasma group, p=0.62).<br><br>There was no difference in total alveolar protein between groups (129.7 LR group vs 123.4 mg/ml plasma group, p=0.11).<br><br>Resuscitation with plasma was associated with a decrease in the expression of inflammatory genes in the lungs.
Chang et al.[74]	2018	Rats cecal ligation and puncture.<br><br>normal saline vs fresh frozen plasma resuscitation	Mortality<br><br>Biomarker levels	The use of FFP resulted in a significant increase in survival rates, with 57% in the FFP group compared to 14% in the control group at 48 hours. Additionally, FFP was associated with reduced levels of norepinephrine (3.8 ng/mL in the FFP group vs 8.9 ng/mL in the control group), interleukin-6 (IL-6) (3.8 ng/mL in the FFP group vs 18.7 ng/mL in the control group), and syndecan-1 (21.8 ng/mL in the FFP group vs 31.0 ng/mL in the control group).

Lactated ringers (LR), Fresh frozen plasma (FFP), Interleukin-6 (IL-6)

Supplemental Table 4: Clinical Studies of Plasma in Sepsis

Authors	Year	Population	N	Design	Outcome	Result
Dietrich et al. [79]	2022	Septic shock patients admitted to the ICU	261	Retrospective chart review<br><br>Comparing FFP transfusion within the first 48 hours of resuscitation transfusion within	Mortality<br><br>IV fluids, ICU stay and ventilator days.	100 patients (38.3%) received FFP within the first 48 hours<br><br>FFP had no significant effect on 30-day or 90-day mortality, amount of fluids transfused, ICU length of stay, or ventilator days. There was no significant harm or benefit.
El- Nawawy et al.[78]	2021	Pediatric intensive care patients with sepsis or septic shock with no overt DIC.	80	Open label RCT of plasma, low-dose heparin and tranexamic acid versus standard care	Mortality<br><br>DIC	Mortality in the intervention group was 10% and standard of care group 50% ($p<0.001$)<br><br>DIC was significantly higher in the standard of care group compared to the intervention group (45 % vs 10 % respectively, $p<0.0001$)
Straat et al[77]	2015	Critically ill patients who are coagulopathic but non-bleeding	33	Prospective RCT sub- study of FFP in non-bleeding critically ill patients	Biomarker levels (TNF-a and syndecan-1)	45% of patients had sepsis. Change in concentration before versus after FFP transfusion: 1) TNF-α (11.3 pg/ml vs 2.3 pg/ml, $p<0.01$) and syndecan-1 levels (675 vs 565 , $p<0.01$)

Intensive Care Unit (ICU), Intravenous (IV), Fresh Frozen Plasma (FFP), Disseminated Intravascular Coagulopathy (DIC), Tumor necrosis factor alpha (TNF-α), Randomized Clinical Trial (RCT)
